# Pericyte Loss in Diseases

**DOI:** 10.3390/cells12151931

**Published:** 2023-07-26

**Authors:** Pengfei Li, Hongkuan Fan

**Affiliations:** Department of Pathology and Laboratory Medicine, Medical University of South Carolina, Charleston, SC 29425, USA; lippe@musc.edu

**Keywords:** perictyes, vascular leak, diabetes, blood–brain barrier

## Abstract

Pericytes are specialized cells located in close proximity to endothelial cells within the microvasculature. They play a crucial role in regulating blood flow, stabilizing vessel walls, and maintaining the integrity of the blood–brain barrier. The loss of pericytes has been associated with the development and progression of various diseases, such as diabetes, Alzheimer’s disease, sepsis, stroke, and traumatic brain injury. This review examines the detection of pericyte loss in different diseases, explores the methods employed to assess pericyte coverage, and elucidates the potential mechanisms contributing to pericyte loss in these pathological conditions. Additionally, current therapeutic strategies targeting pericytes are discussed, along with potential future interventions aimed at preserving pericyte function and promoting disease mitigation.

## 1. Introduction

Pericytes were first characterized by Eberth and Rouget nearly 150 years ago [1,2,3]. They are specialized cells located within the capillary basement membrane that wrap around endothelial cells in the microcirculation throughout the body [4]. Although a unique pericyte marker has yet to be identified, pericytes are commonly characterized by a combination of markers, including platelet-derived growth factor receptor β (PDGFRβ), the proteoglycan neural glial antigen-2 (NG2), alpha-smooth muscle actin (α-SMA), CD13, CD73, and CD146 [1,2,5,6,7]. It should be noted that the expression of these markers on pericytes is dynamic and tissue dependent [2,8]. Consequently, the distribution and function of pericytes are associated with endothelial barrier properties and exhibit tissue-specific variations [4,8] (Table 1). The developmental origin of pericytes is heterogeneous and remains largely unknown [9]. The origin of pericytes in the lung and liver can be traced to the mesothelium, while the origin of pericytes in the heart was traced to the epicardial mesothelium [9]. The brain and retina harbor the highest density of pericytes, with a pericyte-to-endothelial cell (EC) ratio of 1:1 [2,4,8,10]. These pericytes play a critical role in establishing the blood–brain barrier (BBB) and blood–retinal barrier (BRB), safeguarding brain and retinal cells against potentially harmful blood-derived factors [2,4,8,10]. The typical pericytes in the central nervous system (CNS) are flattened, or elongated, stellate-shaped solitary cells [11]. In the lung, pericytes are indispensable for maintaining pulmonary vasculature function and optimal gas exchange, with a pericyte-to-EC ratio of approximately 1:7–1:9 [8,12]. These cells have a spindle-shaped or stellate morphology and elongated, multibranching cellular processes [13]. However, activated and differentiated lung pericytes also contribute to inflammatory responses and fibrosis [14,15,16]. Renal pericytes, including peritubular pericytes, mesangial cells, and podocytes (pericyte-like cells), are involved in regulating blood ultrafiltration and vascular permeability, with a pericyte-to-EC ratio of about 1:2.5 in the kidney [2,4,17]. Mesangial cells are rounded and compact [11]. Hepatic stellate cells (HSC), the pericytes in the liver, are located between the parenchymal cell plates and the sinusoidal endothelial cells. These cells are characterized by their dendritic structures with cytoplasm filled with fat-storing droplets containing vitamin A and are implicated in extracellular matrix remodeling, vitamin A metabolism, and the recruitment of inflammatory cells [2,18,19,20]. In the liver, the ratio of pericytes to ECs is approximately 1:10 [8,18]. In the heart, pericytes show typical spindle-shaped morphology [21]. The coverage of cardiac pericytes to endothelial cells is about 1:2 to 1:3, and they play a critical role in regulating blood flow, vascular remodeling, and maintaining vascular function [22]. Collectively, pericytes contribute to essential functions in various organs, and alterations in pericyte coverage or density can lead to vascular disturbances and organ dysfunction.

Increasing evidence supports the notion that pericyte loss disrupts vascular homeostasis and contributes to disease progression in diverse conditions, including diabetes, Alzheimer’s disease (AD), amyotrophic lateral sclerosis (ALS), sepsis, stroke, and traumatic brain injury (TBI). This article aims to provide a comprehensive summary of the role and mechanisms of pericyte loss and discuss potential therapies targeting pericytes.

## 2. Pericyte Loss in Diseases

### 2.1. Pericyte Loss in Diabetes

Diabetes is a chronic health condition that causes multiple vascular complications, such as retinopathy and nephropathy [23]. Pericyte loss is an early hallmark of diabetes-associated microvascular diseases and plays a crucial role in the disease progression of various organs including the retina, kidney, brain, and heart [23,24].

#### 2.1.1. Pericyte Loss in Diabetic Retinopathy (DR)

DR, a major complication of diabetes, is the leading cause of blindness worldwide and is characterized by vascular damage in the retina [25,26,27]. Among the vascular cells, pericytes are the earliest to be affected by diabetes, and their loss is a hallmark of diabetic retinopathy, contributing to blood vessel leakage [28]. The loss of pericytes has been detected in the retinas of diabetic patients [29,30,31], as well as in various animal models, including mice [31,32,33], dogs [34,35], hamsters [36], and rats [37,38].

The precise mechanisms underlying pericyte loss in diabetic retinopathy are not yet fully understood. One hypothesis suggests that pericyte death via apoptosis is involved, as confirmed by studies in both patients and animal models of DR [30,32,39,40,41,42]. Mechanically, factors such as high-glucose [40,43], oxidative stress [44], advanced glycation end products [45,46], TNF-α [47], and IL-1β [48] have been shown to induce apoptosis in retinal pericytes in vitro and/or in vivo. Additionally, the migration of retinal pericytes may contribute to pericyte loss in DR, regulated by the Ang-Tie system and autophagy processes [49,50]. Pericyte loss contributes to EC-pericyte dissociation and vascular dysfunction as retinal capillary pericytes are critical to maintaining EC-pericyte contacts and the integrity of vascular barrier function via secretion of sphingosine 1-phosphate [51,52]. Therefore, preventing the loss of retinal pericytes would be beneficial. Pericytes derived from adipose-derived stem cells (ASCs) showed protective effects against capillary loss in the retina in a murine model of DR [53]. Additionally, human pericyte-like ASCs have demonstrated the ability to protect human retinal endothelial cells in an in vitro model of DR [54]. Therefore, pericyte/pericyte-like cell-targeting therapies or cell implantation of pericytes/pericyte-like cells hold promise for the treatment of DR and warrant further investigation.

#### 2.1.2. Pericyte Loss in Diabetic Nephropathy (DN)

As a common complication of diabetes, DN is characterized by proteinuria, microvascular damage, and the disruption of glomeruli and the tubular system [4,23,55,56]. It significantly impacts the quality of life of diabetic patients and is the leading cause of end-stage renal disease [23,56]. Renal pericyte-like cells, including peritubular pericytes, mesangial cells, and podocytes, are susceptible to oxidative stress induced by high glucose and play a critical role in the progression of DN [4,23]. 

Several hallmark features of DN, such as peritubular capillary rarefaction (PTC) and peritubular fibrosis, mesangial and glomerular hypertrophy, and podocyte injury, are closely associated with renal dysfunction [4,55,57,58,59]. Peritubular pericytes are crucial for maintaining the integrity of peritubular capillaries, as the loss of pericytes can accelerate PTC rarefaction [58,60,61]. Furthermore, the migration of peritubular pericytes away from the capillaries and their transformation into myofibroblasts are potential mechanisms underlying PTC and peritubular fibrosis [4,62,63]. However, the precise role and underlying mechanisms of peritubular pericytes in DN remain largely unknown. Mesangial cells, comprising approximately 30% of glomerular cells, undergo hypertrophy in the early stages of DN [4,23,64,65]. Increased mTOR activity may contribute to mesangial cell hypertrophy under high glucose conditions [4,65]. Moreover, diabetic rats and mice exhibit glomerular cell loss and apoptosis, which are associated with albuminuria and renal dysfunction [66,67]. Mechanistically, elevated levels of urinary miR-15b-5p have been observed in diabetic patients and db/db mice and contribute to high glucose-induced mesangial cell apoptosis [68]. Additionally, serum levels of Angpt2 were increased in diabetic patients and db/db mice, and the Angpt2/miR-33-5p/SOCS5 signaling pathway has been implicated in mesangial cell apoptosis under high glucose conditions [69]. 

Podocytes, which are pericyte-like cells, play a crucial role in the progression of DN [4,23,70], and podocyte injury is a hallmark of both DN and non-diabetic kidney diseases [71,72]. The loss of podocytes serves as an early pathological marker and contributes to proteinuric glomerulopathies in DN [70,71,73]. The number of podocytes is decreased in both type 1 and type 2 diabetic patients [74,75], and podocyte loss has also been observed in diabetic mice [76,77] and rats [78,79]. Podocyte apoptosis is the most common mechanism of podocyte loss and has been extensively documented [71,80,81,82]. Mechanistically, the accumulation of harmful factors such as reactive oxygen species (ROS), advanced glycation end products, miRNAs, and angiotensin II, along with the activation of signaling pathways, including p53, mTOR, and Notch, are involved and contribute to DN-induced podocyte apoptosis [71,72,83]. Several other pathways have been implicated in podocyte loss in DN, including autophagy, mitotic catastrophe, anoikis, necrosis, and pyroptosis, which have been comprehensively discussed by Jiang et al. [71]. VEGF-A, primarily produced by podocytes, is necessary for the survival of glomerular endothelial cells [84,85]. The loss of podocyte-derived VEGF-A results in EC dysfunction and disrupts the glomerular filtration barrier [84,86]. Therefore, podocyte loss has a significant impact on the dysfunction of EC and the glomerular filtration barrier. 

#### 2.1.3. Brain Pericyte Loss in Diabetes

Diabetes can induce damage that leads to dysfunction of the BBB and cognitive decline in both patients and experimental models [87,88,89,90]. Furthermore, diabetic patients have a higher risk of developing dementia-related diseases such as stroke and Alzheimer’s disease (AD) [91,92]. In the brain, diabetes-related complications are characterized by pericyte loss, increased BBB permeability, and neuronal dysfunction [87,89,93]. Reduced numbers of brain pericytes have been reported in diabetic patients [23,94], as well as in animal models of diabetes, including mice and rats [95,96,97]. In vitro studies have shown that oxidative stress induced by high glucose can lead to apoptosis in cultured brain pericytes [98,99,100,101]. The activity of mitochondrial carbonic anhydrases was believed to induce brain pericyte loss in diabetic mice as the inhibition of mitochondrial carbonic anhydrases activity can reduce oxidative stress and prevent pericyte dropout [96]. However, the exact mechanisms and in vivo processes underlying brain pericyte loss in diabetes require further investigation.

#### 2.1.4. Cardiac Pericyte Loss in Diabetes 

Cardiovascular disease (CVD) is a significant complication of diabetes and is the leading cause of heart failure or mortality in diabetic patients [102,103,104,105]. Pericytes play a crucial role, particularly in the early stages of diabetes-associated CVD, including myocardial and interstitial fibrosis [105,106,107]. The loss of pericytes has been demonstrated in the hearts of diabetic patients and diabetic pigs [108]. Additionally, studies by Tu et al. showed a reduction in the number of cardiac pericytes and microvascular coverage in diabetic mice [24]. The overexpression of thymosin beta 4 has the ability to mitigate cardiac pericyte loss in diabetic pigs, providing a potential therapeutic approach for diabetes-associated CVD [108]. However, the specific underlying mechanisms of cardiac pericyte loss in diabetes remain unclear and require further investigation.

In summary, pericyte loss is closely associated with various complications of diabetes and significantly contributes to disease development. Further research is needed to gain a better understanding of the underlying mechanisms involved and to explore novel therapeutic strategies targeting pericytes.

### 2.2. Pericyte Loss in Aging and Neurodegenerative Diseases 

#### 2.2.1. Pericyte Loss in Alzheimer’s Disease

AD is the most prevalent neurodegenerative disorder characterized by cognitive impairment, an accumulation of amyloid β-peptide (Aβ), BBB dysfunction, and neuroinflammation [109,110,111]. Pericytes play a critical role in AD, as their deficiency in mouse models of AD accelerates BBB breakdown and increases Aβ accumulation in the brain [112]. Pericyte loss has been reported in various regions of AD patients’ brains, including the white matter [113,114], precuneus [115], cortex [112,116], hippocampus [7,116], and retina [117]. Similarly, reduced pericyte numbers have been observed in the cortex [118,119], hippocampus [7,119], and retina [120] of AD mice. In the retina, the activation of inflammation appears to contribute to pericyte loss as an association between NF-κB p65 phosphorylation levels and vascular PDGFRβ expression was observed in AD mice [120]. Apoptosis is believed to contribute to pericyte loss, as pericyte apoptosis has been identified in the retina and hippocampus of AD patients [7,117]. In vitro studies have shown that Aβ stimulation induces apoptosis in cultured brain pericytes [7,119]. Mechanistically, decreased miR-181a levels and enhanced Fli-1 expression may contribute to pericyte loss and apoptosis in AD [7,119]. A reduced miR-181a expression has been observed in AD mice, but the overexpression of miR-181a can mitigate pericyte loss, improve BBB function, and decrease Aβ accumulation [119]. Furthermore, miR-181a inhibits Aβ-induced pericyte apoptosis in murine brain cell cultures [119]. Our recent study suggests that Fli-1 expression is increased in postmortem brains from AD donors and in a mouse model of AD known as 5xFAD. The inhibition of Fli-1 via antisense oligonucleotide Fli-1 Gapmer decelerates pericyte loss, reduces inflammatory response, ameliorates cognitive deficits, improves BBB function, and decreases Aβ deposition [7]. In addition, Fli-1 Gapmer treatment protects against Aβ-induced apoptosis in human brain pericytes in vitro [7]. Aβ-evoked pericyte-mediated constriction of the cerebral capillary bed contributes to the reduction in cerebral blood flow during AD [121]. Moreover, EC-pericyte contacts are important to control cerebral blood flow and promote EC survival via pericyte-derived VEGF [122]. The loss of pericytes leads to increased VEGF expression in EC, which may be a compensatory signaling pathway [123]. Thus, pericyte loss may contribute to reduced cerebral blood flow and EC dysfunction. The implantation of pericytes derived from mesenchymal stem cells has been shown to enhance cerebral blood flow and reduce Aβ levels in a mouse model of AD, suggesting that cell-based therapies targeting pericytes/pericyte-like cells may hold promise in the prevention and treatment of AD [124].

#### 2.2.2. Pericyte Loss in Amyotrophic Lateral Sclerosis (ALS)

ALS, a fatal neurodegenerative disorder, is characterized by blood–spinal cord barrier dysfunction and the progressive degeneration of motor neurons [125,126,127,128]. Recent studies have highlighted the important role of pericytes in ALS [129]. Decreased pericyte coverage or number has been observed in the ventral horn and spinal cord of ALS patients, which correlates with vascular disruption [130,131]. Furthermore, a loss of pericytes in the choroid plexus has been detected in patients with ALS, coupled with a deregulation of the blood–cerebrospinal fluid (CSF) barrier [132]. In a murine model of ALS, reduced pericyte coverage in spinal cord capillaries has also been demonstrated [133]. Interestingly, the administration of adipose-derived pericytes has shown promising results in ALS mice, extending their survival and increasing antioxidant enzymes in the brain [134]. These findings suggest that pericytes may represent a novel potential cell therapy for treating ALS, although further studies are needed to fully understand pericyte loss in ALS and its implications for disease progression.

Overall, pericyte loss in aging and neurodegenerative diseases poses a significant challenge that can have negative effects on brain health. Advancing our understanding of the underlying mechanisms of pericyte loss and developing new treatments to prevent or reverse this process are important areas of future research.

### 2.3. Pericyte Loss in Infectious Diseases

#### 2.3.1. Pericyte Loss in Sepsis

Sepsis is a life-threatening condition caused by a microbial infection resulting in organ dysfunction and failure. It is characterized by a systemic inflammatory response and microvascular dysfunction [135,136]. Recent studies have highlighted the role of dysfunctional pericytes in sepsis-induced microvascular dysfunction, which serves as a hallmark of severe sepsis and septic shock [15,137]. Research by Nishioku et al. demonstrated the detachment of pericytes from the basal lamina in the hippocampus of LPS-treated mice [138]. The detachment of pericytes may contribute to sepsis-induced BBB dysfunction [139] as pericytes control vascular permeability in the brain [140]. Pericyte loss has also been observed in the lungs and hearts of LPS-treated mice, although this loss is not caused by apoptosis [141]. Reduced pericyte coverage in mesenteric microvessels has been demonstrated in both cecal ligation and puncture (CLP) and LPS-induced septic rats [142]. In a previous study, we showed a reduction in pericyte density in the lungs and kidneys of CLP-induced septic mice, suggesting pericyte pyroptosis as a potential mechanism for this loss [15]. Our findings indicate that an increased expression of Fli-1 in lung pericytes may contribute to pericyte pyroptosis and the knockout of Fli-1 in pericytes attenuates lung pericyte loss, vascular leak, and mortality in a murine model of sepsis [15]. Moreover, angiopoietin-2, which is increased in septic patients, has been implicated in pericyte loss, as endothelial angiopoietin-2 overexpressed mice displayed significant pericyte loss [143,144]. Furthermore, the disruption of Sirt3/angiopoietins/Tie-2 and HIF-2α/Notch3 pathways is also critical for LPS-induced lung pericyte loss [141]. Importantly, pericyte transplantation has been shown to reduce pericyte loss and increase the survival rate in septic rats [142]. In addition, microvesicles derived from pericytes have improved pulmonary function in a rat model of sepsis [145]. These findings suggest that therapeutic strategies targeting pericytes for sepsis hold promise, and a further understanding of the underlying mechanisms of pericyte dysfunction and loss in sepsis is needed. 

#### 2.3.2. Pericyte Loss in HIV

The neurocognitive disorder is a major complication of HIV as the virus enters the brain shortly after infection, leading to inflammation and BBB disruption [146,147]. In vitro studies have demonstrated that cultured brain pericytes can be infected by HIV, resulting in enhanced production of inflammatory mediators and disruption of endothelial barrier properties [148,149]. Furthermore, evidence from in vivo studies, including HIV patients and mouse models of HIV, has shown that brain pericytes can be infected by HIV [150,151,152]. Following HIV infection, a reduction in pericyte coverage has been observed in the brains of HIV patients [150,153,154]. Similar pericyte loss has also been detected in the brains of mouse models of HIV and SIV-infected macaques [153,154]. It has been suggested that the higher concentration of PDGF-BB induced by HIV Tat via the activation of mitogen-activated protein kinases and nuclear factor-κB pathways may drive HIV-induced pericyte loss in the brain [153,155]. However, the role of pericytes in HIV has not been extensively examined. A better understanding of pericyte dysfunction and loss in the context of HIV may provide opportunities for the development of novel therapeutics.

#### 2.3.3. Pericyte Loss in COVID-19

COVID-19 is caused by the severe acute respiratory syndrome coronavirus 2 (SARS-CoV-2) and affects various organs, including the heart, brain, and lungs [156,157,158,159]. Cardiac pericytes, which express high levels of angiotensin-converting enzyme 2 (ACE-2), the main receptor for SARS-CoV-2, are major targets for viral infection [160,161,162]. The infection of pericytes via SARS-CoV-2 contributes to cardiac complications associated with COVID-19, such as thrombosis, inflammation, and hemodynamic disturbances [163]. Studies have shown a significant loss of pericyte coverage in the heart capillaries of hamsters infected with SARS-CoV-2 [164]. Additionally, SARS-CoV-2 can infect cardiac pericytes, and its spike protein may induce pericyte dysfunction via CD147 receptor-mediated signaling pathway, leading to microvascular injury [157]. Brain pericytes, which also express ACE-2, are susceptible to SARS-CoV-2 infection, potentially driving inflammation and vascular dysfunction [158,165,166]. Patients with COVID-19 have shown lower levels of the pericyte marker PDGFRβ in their cerebrospinal fluid [158]. SARS-CoV-2 spike protein has been found to deregulate vascular and immune functions in brain pericytes [167], while the SARS-CoV-2 envelope protein has been shown to induce brain pericyte death in vitro [168]. In the lung, pericytes were infected by SARS-CoV-2 and are detached from pulmonary capillary endothelium in COVID-19 patients [159,169]. However, the underlying mechanisms of pericyte loss in COVID-19 remain largely unknown, and further studies are needed to investigate the role of pericytes, particularly in long COVID-19 [170]. 

Overall, the loss of pericytes in infectious diseases can have significant negative effects on patient outcomes. Understanding the mechanisms underlying pericyte loss in these diseases is crucial for the development of new treatments that can prevent or reverse this process and improve patient outcomes. Further research is needed to gain a better understanding of the role of pericytes in infectious diseases and to explore novel therapeutic approaches that can target these cells.

### 2.4. Pericyte Loss in Brain Injury

#### 2.4.1. Pericyte Loss in Stroke

Stroke, a leading cause of death and disability worldwide, is associated with pericyte dysfunction and BBB disruption [171]. Pericytes play a critical role in regulating inflammation, angiogenesis and BBB function during stroke [171,172]. A rapid reduction in brain pericyte number and coverage has been observed in human stroke cases as well as in experimental stroke models, including mice and rats, following ischemic damage [113,173,174,175,176]. Pericyte apoptosis and autophagy have been detected in the brain from murine models of stroke, which may contribute to pericyte loss and BBB disruption [174,175]. The loss of regulator of G protein signaling 5 (RGS5) has been associated with increased pericyte number and improved BBB function in a mouse model of stroke, suggesting a role for RGS5 in brain pericyte loss during stroke [177]. Additionally, the inhibition of Sema3E/PlexinD1 signaling has been shown to increase pericyte number and enhance blood–brain barrier integrity in aged rats with stroke, further implicating this signaling pathway in brain pericyte loss [176]. Moreover, the deletion of hypoxia-inducible factors (HIF)-1 in pericytes has been found to prevent brain pericyte apoptosis and reduce vascular permeability in mice with stroke, indicating the involvement of pericyte HIF-1 in stroke-induced pericyte apoptosis [175]. In addition to maintaining BBB function by themselves, pericytes also promote the physiological functions of other BBB components including endothelial cells, basal lamina, and astrocytes [178]. For example, pericytes regulate aquaporin-4 polarization in mouse cortical astrocytes [179]. Furthermore, angiopoietin-1 secreted by pericytes mediates tight junction induction via the activation of Tie-2, an angiopoietin-1 receptor on EC [178,180,181]. Therefore, restoration of pericyte coverage may improve BBB support and promote reperfusion after stroke [182]. Gaining more insights into the role of pericytes in stroke could facilitate the development of novel therapeutic approaches for stroke treatment [172].

#### 2.4.2. Pericyte Loss in Traumatic Brain Injury (TBI)

TBI, caused by an external force, is the major cause of mortality and disability, particularly in young individuals [183]. The secondary injury following TBI involves oxidative stress, inflammation, and the production of matrix metalloproteinases (MMPs), which contribute to BBB dysfunction [184,185,186]. Recent studies have highlighted pericyte degeneration as a significant factor in TBI, leading to regional microcirculatory hypoperfusion and increased BBB permeability [187,188]. A decline in pericyte markers has been observed in brain specimens from human TBI cases and in a mouse model of repetitive mild TBI up to 12 months post-injury [189]. Additionally, rapid pericyte loss in the acute phase of TBI has been documented in the brains of mice with TBI [188,189,190,191,192]. Brain pericyte apoptosis has been detected in a mouse model of TBI, suggesting that pericyte loss during TBI may be attributed to apoptosis [193]. It has been found that the inhibition of the TNF-α/NF-κB/iNOS axis can reverse pericyte loss, improve pericyte function, and enhance microcirculation perfusion after TBI [188]. This indicates the potential contribution of the TNF-α/NF-κB/iNOS axis to pericyte loss in TBI. Consequently, the development of treatments that can prevent or reverse pericyte degeneration holds promise for the management of TBI and the secondary injuries that follow.

Overall, understanding the mechanisms of pericyte loss in these conditions is crucial for developing new treatments that can prevent or reverse this process and improve patient outcomes. 

## 3. Methods to Determine Pericyte Loss

As there is no universally recognized marker for pericytes, and pericyte markers can vary depending on the specific tissues, it becomes crucial to employ various methods for detecting and assessing pericyte changes. In this section, we will explore several approaches commonly used to determine and evaluate alterations in pericytes.

### 3.1. Immunohistochemistry

One commonly utilized method to assess pericyte changes is immunohistochemistry, which enables the visualization and quantification of pericytes within tissues. This technique involves staining tissue samples with specific antibodies targeting pericyte markers and/or vascular markers (Table 2). In studies focusing on the brain, pericytes have been identified via the immunostaining of various markers, such as PDGFR-β [95,97,118,191], CD13 [7], NG2 [97], NG2+CD31 [153], CD13+CD31 [150], PDGFR-β+CD31 [194], a-SMA+laminin [138], PDGFR-β+laminin [195], PDGFR-β+lectin [196], CD13+lectin [119,192], and desmin+lectin [197]. In retinal investigations, pericytes have been detected via the immunostaining of markers such as α-SMA [42], NG2 [33,38,42], and PDGFR-β+lectin [117,120] allowing for the determination of pericyte number and coverage. Lung pericytes have been characterized by the immunostaining of NG2+IB4 [141] and Foxd1+CD31 [15]. Similarly, cardiac pericyte changes have been observed via the immunostaining of NG2 [108], NG2+IB4 [24,141], NG2+isolectin [164], and NG2+PDGFR-β [157]. In the case of kidneys, immunostaining with antibodies specific to WT1 [78,81,82] has been employed to detect changes in renal podocytes, which are pericyte-like cells in the kidneys. Additionally, immunohistochemical staining of CD13+laminin has been utilized to assess pericyte changes in the spinal cord [133]. Collectively, combining pericyte markers with vascular markers remains the predominant approach for immunohistochemical detection of pericyte density and coverage in various tissues.

### 3.2. Electron Microscopy

Another valuable method utilized for assessing pericyte loss is electron microscopy. This technique involves capturing high-resolution images of tissue samples using an electron microscope, enabling the visualization of cellular structures with great detail. Electron microscopy is particularly effective in visualizing pericytes and detecting alterations in their morphology, such as detachment from blood vessels, shrinkage, or loss of cellular organelles. For instance, in studies involving diabetic mice, transmission electron microscopy (TEM) has been employed to detect changes in the morphology and density of renal podocytes [76]. Scanning electron microscopy has been utilized to observe the detachment of brain pericytes from the capillary wall [198]. Additionally, TEM has proven useful in visualizing the interaction between pericytes and endothelium [199]. Furthermore, TEM has been widely applied to identify pericyte changes in various tissues affected by different diseases [174,200,201,202].

### 3.3. Live Animal Imaging Techniques

Live animal imaging techniques, such as intravital microscopy or two-photon microscopy, play a crucial role in determining pericyte changes in vivo. These advanced methods allow for the visualization of live tissues using fluorescently labeled antibodies or cells. By employing live animal imaging, researchers gain real-time, dynamic information about pericyte behavior and their interactions with other cells. For instance, confocal intravital microscopy has been successfully utilized to study the interactions between neutrophils and pericytes in vivo [203]. Furthermore, intravital microscopy has been employed to investigate the dynamic interactions of endothelial cells and pericytes [204,205]. Changes in pericytes can be detected via alterations in fluorescent signals or by observing modifications in the morphology or behavior of fluorescently labeled cells. Two-photon microscopy has also been widely used to study dynamic changes, spatial distribution, density, and the subsets of pericytes [206,207,208,209,210]. These live animal imaging techniques are invaluable for investigating the role and mechanisms of pericytes in regulating vascular function. Additionally, they provide crucial insights into the dynamic interactions between pericytes and surrounding cells during different stages of diseases. These techniques hold great promise for future research endeavors in this field. 

### 3.4. Other Techniques

Additional techniques, such as Western blot and ELISA, have been employed as supportive methods to determine pericyte changes by quantifying protein levels of pericyte markers in tissues and cerebrospinal fluid (CSF). Western blot analysis has revealed decreased expression levels of pericyte markers in the lung of septic mice, corroborating the observations of lung pericyte loss reported in immunohistochemistry studies [15,141]. ELISA measurements of PDGFR-β expression have shown correlations with pericyte numbers in the brain white matter of stroke and Alzheimer’s disease patients [113]. Furthermore, ELISA-based detection of soluble PDGFRβ levels in the CSF serves as an indicator of brain pericyte injury, often associated with blood–brain barrier breakdown [211,212,213]. Recent advancements in molecular biology techniques, such as single-cell sequencing and RNA sequencing, have revolutionized the identification and characterization of pericytes at the molecular level. These cutting-edge techniques offer valuable insights into the gene expression patterns of pericytes, the subsets of pericytes, and how they undergo changes in response to diseases or injuries [214,215,216,217,218]. By employing these techniques, researchers can identify subtypes of pericytes and discern their distinct roles in various diseases. 

In summary, the detection of pericyte loss is crucial for the diagnosis and treatment of numerous diseases. There are several methods available to determine pericyte changes, including immunohistochemistry, electron microscopy, live imaging, and molecular biology techniques. The selection of the appropriate method depends on the specific research question, the type of tissue being studied, and the availability of resources. Combining multiple techniques can offer a more comprehensive understanding of the role and dynamic changes of pericytes in different disease contexts. By employing a multidimensional approach, researchers can gain valuable insights into the complex behavior and functions of pericytes, facilitating advancements in disease diagnosis, treatment, and therapeutic interventions.

## 4. Conclusions

In conclusion, the detection of pericyte loss or dysfunction has been established in various diseases, and their contribution to pathological progression is well recognized. Pericytes exhibit multifunctional properties, offering potential avenues for therapeutic interventions in conditions involving inflammation, fibrosis, angiogenesis, and vascular dysfunction [171,182,219]. Preclinical studies have demonstrated the efficacy of treatments targeting pericytes, such as modulating gene expression or implanting pericytes, in animal models of sepsis [15], stroke [175], diabetic retinopathy [53], Alzheimer’s disease [124], and amyotrophic lateral sclerosis [134]. Future therapeutic approaches targeting pericytes/pericyte-like cells can be explored from several of the following angles: (1) modulation of signaling pathways in pericytes or surrounding cells that contribute to pericyte loss or dysfunction; (2) reduction in detrimental factors that induce damage and degeneration in pericytes; (3) implementation of pericytes or specific subpopulations derived from various organ origins for cell-based therapies; (4) utilization of multipotential stem cells to generate pericytes/pericyte-like cells for implantation; (5) utilization of exosomes derived from healthy or modified pericytes. Taken together, pericytes represent a promising target for the development of novel therapeutic treatments. Further research and advancements in understanding pericyte biology and its interactions within the microenvironment will enhance our ability to harness the therapeutic potential of pericytes, leading to improved clinical outcomes in a wide range of diseases.

## Figures and Tables

**Table 1:** The function and coverage of pericytes in organs.

Tissue	Function	Pericytes/EC Ratio
Brain	Maintaining BBB function, the recruitment of inflammatory cells, regulating cerebral blood flow, Aβ clearance, and inflammation	1:1
Retina	Maintaining BRB function, regulating Aβ clearance, and inducing immune responses	1:1
Lung	Regulating inflammatory response, maintaining the pulmonary vasculature, and optimal gas exchange	1:7–1:9
Kidney	Maintaining the integrity of peritubular capillaries, regulating blood ultrafiltration and vascular permeability	1:2.5
Liver	Remodeling of the ECM, vitamin A metabolism, and the recruitment of inflammatory cells	1:10
Heart	Regulating blood flow, vascular remodeling, and myocardial and interstitial fibrosis	1:2–1:3

Aβ: amyloid β-peptide; BBB: Blood-brain barrier; BRB: blood–retinal barrier; EC: endothelial cells; ECM: extracellular matrix.

**Table 2:** Markers used to detect pericytes in different tissues via immunohistochemistry.

Tissue	Disease	Pericyte and/or Vascular Markers
Brain	Diabetes	PDGFR-β [95,97], NG2 [97]
Brain	Sepsis	a-SMA+laminin [138]
Brain	HIV	NG2+CD31 [153], CD13+CD31 [150]
Brain	AD	CD13 [7], PDGFR-β [118], CD13+lectin [119], PDGFR-β+lectin [196]
Brain	MS	PDGFR-β+ laminin [195], Desmin+lectin [197]
Brain	Brain metastases	PDGFR-β+CD31 [194]
Brain	TBI	PDGFR-β [191], CD13+lectin [192]
Retina	Diabetes	a-SMA [42], NG2 [33,38,42]
Retina	AD	PDGFR-β+lectin [117,120]
Lung	Sepsis	NG2+IB4 [141], Foxd1+CD31 [15]
Heart	Diabetes	NG2 [108], NG2+IB4 [24]
Heart	Sepsis	NG2+IB4 [141]
Heart	COVID-19	NG2+ isolectin [164], NG2+ PDGFR-β [157]
Kidney	Diabetes	WT1 [78,81,82]
Spinal cord	ALS	CD13+laminin [133]

AD: Alzheimer’s disease; MS: multiple sclerosis; TBI: traumatic brain injury; ALS: amyotrophic lateral sclerosis.

## Data Availability

No new data were created.

## References

[1] Attwell D., Mishra A., Hall C.N., O’Farrell F.M., Dalkara T. (2016). What is a pericyte?. J. Cereb. Blood Flow. Metab..

[2] Bergers G., Song S. (2005). The role of pericytes in blood-vessel formation and maintenance. Neuro Oncol..

[3] Dore-Duffy P. (2008). Pericytes: Pluripotent cells of the blood brain barrier. Curr. Pharm. Des..

[4] Ferland-McCollough D., Slater S., Richard J., Reni C., Mangialardi G. (2017). Pericytes, an overlooked player in vascular pathobiology. Pharmacol. Ther..

[5] Armulik A., Genove G., Betsholtz C. (2011). Pericytes: Developmental, physiological, and pathological perspectives, problems, and promises. Dev. Cell.

[6] Li P., Wu Y., Goodwin A.J., Halushka P.V., Wilson C.L., Schnapp L.M., Fan H. (2021). Generation of a new immortalized human lung pericyte cell line: A promising tool for human lung pericyte studies. Lab. Investig..

[7] Li P., Wu Y., Hamlett E.D., Goodwin A.J., Halushka P.V., Carroll S.L., Liu M., Fan H. (2022). Suppression of Fli-1 protects against pericyte loss and cognitive deficits in Alzheimer’s disease. Mol. Ther..

[8] Zhang Z.S., Zhou H.N., He S.S., Xue M.Y., Li T., Liu L.M. (2020). Research advances in pericyte function and their roles in diseases. Chin. J. Traumatol..

[9] Yamazaki T., Mukouyama Y.S. (2018). Tissue Specific Origin, Development, and Pathological Perspectives of Pericytes. Front. Cardiovasc. Med..

[10] Shepro D., Morel N.M. (1993). Pericyte physiology. FASEB J..

[11] Armulik A., Abramsson A., Betsholtz C. (2005). Endothelial/pericyte interactions. Circ. Res..

[12] Garrison A.T., Bignold R.E., Wu X., Johnson J.R. (2023). Pericytes: The lung-forgotten cell type. Front. Physiol..

[13] Wilson C.L., Stephenson S.E., Higuero J.P., Feghali-Bostwick C., Hung C.F., Schnapp L.M. (2018). Characterization of human PDGFR-beta-positive pericytes from IPF and non-IPF lungs. Am. J. Physiol. Lung Cell Mol. Physiol..

[14] Hung C.F., Mittelsteadt K.L., Brauer R., McKinney B.L., Hallstrand T.S., Parks W.C., Chen P., Schnapp L.M., Liles W.C., Duffield J.S. (2017). Lung pericyte-like cells are functional interstitial immune sentinel cells. Am. J. Physiol. Lung Cell Mol. Physiol..

[15] Li P., Zhou Y., Goodwin A.J., Cook J.A., Halushka P.V., Zhang X.K., Wilson C.L., Schnapp L.M., Zingarelli B., Fan H. (2018). Fli-1 Governs Pericyte Dysfunction in a Murine Model of Sepsis. J. Infect. Dis..

[16] Sun Y., Sun W., Yang N., Liu J., Tang H., Li F., Sun X., Gao L., Pei F., Liu J. (2019). The effect of core fucosylation-mediated regulation of multiple signaling pathways on lung pericyte activation and fibrosis. Int. J. Biochem. Cell Biol..

[17] Betsholtz C. (2004). Insight into the physiological functions of PDGF through genetic studies in mice. Cytokine Growth Factor. Rev..

[18] Hellerbrand C. (2013). Hepatic stellate cells—The pericytes in the liver. Pflugers Arch..

[19] Sato M., Suzuki S., Senoo H. (2003). Hepatic stellate cells: Unique characteristics in cell biology and phenotype. Cell Struct. Funct..

[20] Knittel T., Dinter C., Kobold D., Neubauer K., Mehde M., Eichhorst S., Ramadori G. (1999). Expression and regulation of cell adhesion molecules by hepatic stellate cells (HSC) of rat liver: Involvement of HSC in recruitment of inflammatory cells during hepatic tissue repair. Am. J. Pathol..

[21] Avolio E., Rodriguez-Arabaolaza I., Spencer H.L., Riu F., Mangialardi G., Slater S.C., Rowlinson J., Alvino V.V., Idowu O.O., Soyombo S. (2015). Expansion and characterization of neonatal cardiac pericytes provides a novel cellular option for tissue engineering in congenital heart disease. J. Am. Heart Assoc..

[22] Su H., Cantrell A.C., Zeng H., Zhu S.H., Chen J.X. (2021). Emerging Role of Pericytes and Their Secretome in the Heart. Cells.

[23] Warmke N., Griffin K.J., Cubbon R.M. (2016). Pericytes in diabetes-associated vascular disease. J. Diabetes Complicat..

[24] Tu Y., Li Q., Zhou Y., Ye Z., Wu C., Xie E., Li Y., Li P., Wu Y., Guo Z. (2022). Empagliflozin inhibits coronary microvascular dysfunction and reduces cardiac pericyte loss in db/db mice. Front. Cardiovasc. Med..

[25] Yau J.W., Rogers S.L., Kawasaki R., Lamoureux E.L., Kowalski J.W., Bek T., Chen S.J., Dekker J.M., Fletcher A., Grauslund J. (2012). Global prevalence and major risk factors of diabetic retinopathy. Diabetes Care.

[26] Trost A., Bruckner D., Rivera F.J., Reitsamer H.A. (2019). Pericytes in the Retina. Adv. Exp. Med. Biol..

[27] Li Q., Wang M., Li X., Shao Y. (2023). Aging and diabetic retinopathy: Inherently intertwined pathophysiological processes. Exp. Gerontol..

[28] Ren J., Zhang S., Pan Y., Jin M., Li J., Luo Y., Sun X., Li G. (2022). Diabetic retinopathy: Involved cells, biomarkers, and treatments. Front. Pharmacol..

[29] de Oliveira F. (1966). Pericytes in diabetic retinopathy. Br. J. Ophthalmol..

[30] Podesta F., Romeo G., Liu W.H., Krajewski S., Reed J.C., Gerhardinger C., Lorenzi M. (2000). Bax is increased in the retina of diabetic subjects and is associated with pericyte apoptosis in vivo and in vitro. Am. J. Pathol..

[31] Hammes H.P., Lin J., Renner O., Shani M., Lundqvist A., Betsholtz C., Brownlee M., Deutsch U. (2002). Pericytes and the pathogenesis of diabetic retinopathy. Diabetes.

[32] Cheng Y., Peng L., Deng X., Li T., Guo H., Xu C., Fang T., Liu X., Sun B., Chen L. (2021). Prostaglandin F2alpha protects against pericyte apoptosis by inhibiting the PI3K/Akt/GSK3beta/beta-catenin signaling pathway. Ann. Transl. Med..

[33] Jung E., Kim J., Kim C.S., Kim S.H., Cho M.H. (2015). Gemigliptin, a dipeptidyl peptidase-4 inhibitor, inhibits retinal pericyte injury in db/db mice and retinal neovascularization in mice with ischemic retinopathy. Biochim. Biophys. Acta.

[34] Engerman R.L. (1976). Animal models of diabetic retinopathy. Trans. Sect. Ophthalmol. Am. Acad. Ophthalmol. Otolaryngol..

[35] Hammes H.P. (2005). Pericytes and the pathogenesis of diabetic retinopathy. Horm. Metab. Res..

[36] Hammes H.P., Wellensiek B., Kloting I., Sickel E., Bretzel R.G., Brownlee M. (1998). The relationship of glycaemic level to advanced glycation end-product (AGE) accumulation and retinal pathology in the spontaneous diabetic hamster. Diabetologia.

[37] Hammes H.P., Martin S., Federlin K., Geisen K., Brownlee M. (1991). Aminoguanidine treatment inhibits the development of experimental diabetic retinopathy. Proc. Natl. Acad. Sci. USA.

[38] Tang L., Zhang C., Lu L., Tian H., Liu K., Luo D., Qiu Q., Xu G.T., Zhang J. (2022). Melatonin Maintains Inner Blood-Retinal Barrier by Regulating Microglia via Inhibition of PI3K/Akt/Stat3/NF-kappaB Signaling Pathways in Experimental Diabetic Retinopathy. Front. Immunol..

[39] Li W., Yanoff M., Liu X., Ye X. (1997). Retinal capillary pericyte apoptosis in early human diabetic retinopathy. Chin. Med. J..

[40] Romeo G., Liu W.H., Asnaghi V., Kern T.S., Lorenzi M. (2002). Activation of nuclear factor-kappaB induced by diabetes and high glucose regulates a proapoptotic program in retinal pericytes. Diabetes.

[41] Mizutani M., Kern T.S., Lorenzi M. (1996). Accelerated death of retinal microvascular cells in human and experimental diabetic retinopathy. J. Clin. Investig..

[42] Kim Y.H., Park S.Y., Park J., Kim Y.S., Hwang E.M., Park J.Y., Roh G.S., Kim H.J., Kang S.S., Cho G.J. (2012). Reduction of experimental diabetic vascular leakage and pericyte apoptosis in mice by delivery of alphaA-crystallin with a recombinant adenovirus. Diabetologia.

[43] Shin E.S., Huang Q., Gurel Z., Palenski T.L., Zaitoun I., Sorenson C.M., Sheibani N. (2014). STAT1-mediated Bim expression promotes the apoptosis of retinal pericytes under high glucose conditions. Cell Death Dis..

[44] Nie F., Yan J., Ling Y., Liu Z., Fu C., Li X., Qin Y. (2021). Effect of Shuangdan Mingmu capsule, a Chinese herbal formula, on oxidative stress-induced apoptosis of pericytes through PARP/GAPDH pathway. BMC Complement. Med. Ther..

[45] Chen B.H., Jiang D.Y., Tang L.S. (2006). Advanced glycation end-products induce apoptosis involving the signaling pathways of oxidative stress in bovine retinal pericytes. Life Sci..

[46] Sheikpranbabu S., Haribalaganesh R., Gurunathan S. (2011). Pigment epithelium-derived factor inhibits advanced glycation end-products-induced cytotoxicity in retinal pericytes. Diabetes Metab..

[47] Yun J.H., Lee D.H., Jeong H.S., Kim S.H., Ye S.K., Cho C.H. (2022). STAT3 activation in microglia increases pericyte apoptosis in diabetic retinas through TNF-a/AKT/p70S6 kinase signaling. Biochem. Biophys. Res. Commun..

[48] Yun J.H. (2021). Interleukin-1beta induces pericyte apoptosis via the NF-kappaB pathway in diabetic retinopathy. Biochem. Biophys. Res. Commun..

[49] Pfister F., Feng Y., vom Hagen F., Hoffmann S., Molema G., Hillebrands J.L., Shani M., Deutsch U., Hammes H.P. (2008). Pericyte migration: A novel mechanism of pericyte loss in experimental diabetic retinopathy. Diabetes.

[50] Lin W.J., Ma X.F., Zhou H.R., Xu C.Y., Yu X.Y., Hu Y.X., Hao M., Xu Q., Li H.X., Kuang H.Y. (2022). Autophagy Modulates the Migration of Retinal Pericytes Induced by Advanced Glycation End Products. Evid. Based Complement. Altern. Med..

[51] Monickaraj F., McGuire P., Das A. (2018). Cathepsin D plays a role in endothelial-pericyte interactions during alteration of the blood-retinal barrier in diabetic retinopathy. FASEB J..

[52] McGuire P.G., Rangasamy S., Maestas J., Das A. (2011). Pericyte-derived sphingosine 1-phosphate induces the expression of adhesion proteins and modulates the retinal endothelial cell barrier. Arterioscler. Thromb. Vasc. Biol..

[53] Mendel T.A., Clabough E.B., Kao D.S., Demidova-Rice T.N., Durham J.T., Zotter B.C., Seaman S.A., Cronk S.M., Rakoczy E.P., Katz A.J. (2013). Pericytes derived from adipose-derived stem cells protect against retinal vasculopathy. PLoS ONE.

[54] Lupo G., Agafonova A., Cosentino A., Giurdanella G., Mannino G., Lo Furno D., Romano I.R., Giuffrida R., D’Angeli F., Anfuso C.D. (2023). Protective Effects of Human Pericyte-like Adipose-Derived Mesenchymal Stem Cells on Human Retinal Endothelial Cells in an In Vitro Model of Diabetic Retinopathy: Evidence for Autologous Cell Therapy. Int. J. Mol. Sci..

[55] Lenoir O., Jasiek M., Henique C., Guyonnet L., Hartleben B., Bork T., Chipont A., Flosseau K., Bensaada I., Schmitt A. (2015). Endothelial cell and podocyte autophagy synergistically protect from diabetes-induced glomerulosclerosis. Autophagy.

[56] Horton W.B., Barrett E.J. (2021). Microvascular Dysfunction in Diabetes Mellitus and Cardiometabolic Disease. Endocr. Rev..

[57] Khan S.S., Quaggin S.E. (2015). Therapies on the Horizon for Diabetic Kidney Disease. Curr. Diab Rep..

[58] Kida Y. (2020). Peritubular Capillary Rarefaction: An Underappreciated Regulator of CKD Progression. Int. J. Mol. Sci..

[59] Chade A.R. (2017). Small Vessels, Big Role: Renal Microcirculation and Progression of Renal Injury. Hypertension.

[60] Lemos D.R., Marsh G., Huang A., Campanholle G., Aburatani T., Dang L., Gomez I., Fisher K., Ligresti G., Peti-Peterdi J. (2016). Maintenance of vascular integrity by pericytes is essential for normal kidney function. Am. J. Physiol. Renal Physiol..

[61] Kobayashi A., Mugford J.W., Krautzberger A.M., Naiman N., Liao J., McMahon A.P. (2014). Identification of a multipotent self-renewing stromal progenitor population during mammalian kidney organogenesis. Stem Cell Rep..

[62] Lin S.L., Kisseleva T., Brenner D.A., Duffield J.S. (2008). Pericytes and perivascular fibroblasts are the primary source of collagen-producing cells in obstructive fibrosis of the kidney. Am. J. Pathol..

[63] Wu C.F., Chiang W.C., Lai C.F., Chang F.C., Chen Y.T., Chou Y.H., Wu T.H., Linn G.R., Ling H., Wu K.D. (2013). Transforming growth factor beta-1 stimulates profibrotic epithelial signaling to activate pericyte-myofibroblast transition in obstructive kidney fibrosis. Am. J. Pathol..

[64] Schlondorff D. (1987). The glomerular mesangial cell: An expanding role for a specialized pericyte. FASEB J..

[65] Liu L., Hu X., Cai G.Y., Lv Y., Zhuo L., Gao J.J., Cui S.Y., Feng Z., Fu B., Chen X.M. (2012). High glucose-induced hypertrophy of mesangial cells is reversed by connexin43 overexpression via PTEN/Akt/mTOR signaling. Nephrol. Dial. Transplant..

[66] Pesce C., Menini S., Pricci F., Favre A., Leto G., DiMario U., Pugliese G. (2002). Glomerular cell replication and cell loss through apoptosis in experimental diabetes mellitus. Nephron.

[67] Mishra R., Emancipator S.N., Kern T., Simonson M.S. (2005). High glucose evokes an intrinsic proapoptotic signaling pathway in mesangial cells. Kidney Int..

[68] Tsai Y.C., Kuo M.C., Hung W.W., Wu L.Y., Wu P.H., Chang W.A., Kuo P.L., Hsu Y.L. (2020). High Glucose Induces Mesangial Cell Apoptosis through miR-15b-5p and Promotes Diabetic Nephropathy by Extracellular Vesicle Delivery. Mol. Ther..

[69] Tsai Y.C., Kuo P.L., Hung W.W., Wu L.Y., Wu P.H., Chang W.A., Kuo M.C., Hsu Y.L. (2018). Angpt2 Induces Mesangial Cell Apoptosis through the MicroRNA-33-5p-SOCS5 Loop in Diabetic Nephropathy. Mol. Ther. Nucleic Acids.

[70] Barutta F., Bellini S., Gruden G. (2022). Mechanisms of podocyte injury and implications for diabetic nephropathy. Clin. Sci..

[71] Jiang A., Song A., Zhang C. (2022). Modes of podocyte death in diabetic kidney disease: An update. J. Nephrol..

[72] Campbell K.N., Raij L., Mundel P. (2011). Role of angiotensin II in the development of nephropathy and podocytopathy of diabetes. Curr. Diabetes Rev..

[73] Kopp J.B., Anders H.J., Susztak K., Podesta M.A., Remuzzi G., Hildebrandt F., Romagnani P. (2020). Podocytopathies. Nat. Rev. Dis. Primers.

[74] Steffes M.W., Schmidt D., McCrery R., Basgen J.M., International Diabetic Nephropathy Study G. (2001). Glomerular cell number in normal subjects and in type 1 diabetic patients. Kidney Int..

[75] Pagtalunan M.E., Miller P.L., Jumping-Eagle S., Nelson R.G., Myers B.D., Rennke H.G., Coplon N.S., Sun L., Meyer T.W. (1997). Podocyte loss and progressive glomerular injury in type II diabetes. J. Clin. Investig..

[76] Teiken J.M., Audettey J.L., Laturnus D.I., Zheng S., Epstein P.N., Carlson E.C. (2008). Podocyte loss in aging OVE26 diabetic mice. Anat. Rec..

[77] Shi J., Hu Y., Shao G., Zhu Y., Zhao Z., Xu Y., Zhang Z., Wu H. (2023). Quantifying Podocyte Number in a Small Sample Size of Glomeruli with CUBIC to Evaluate Podocyte Depletion of db/db Mice. J. Diabetes Res..

[78] Xue R., Zhai R., Xie L., Zheng Z., Jian G., Chen T., Su J., Gao C., Wang N., Yang X. (2020). Xuesaitong Protects Podocytes from Apoptosis in Diabetic Rats through Modulating PTEN-PDK1-Akt-mTOR Pathway. J. Diabetes Res..

[79] Yang K., Bai Y., Yu N., Lu B., Han G., Yin C., Pang Z. (2020). Huidouba Improved Podocyte Injury by Down-Regulating Nox4 Expression in Rats with Diabetic Nephropathy. Front. Pharmacol..

[80] Susztak K., Raff A.C., Schiffer M., Bottinger E.P. (2006). Glucose-induced reactive oxygen species cause apoptosis of podocytes and podocyte depletion at the onset of diabetic nephropathy. Diabetes.

[81] Niranjan T., Bielesz B., Gruenwald A., Ponda M.P., Kopp J.B., Thomas D.B., Susztak K. (2008). The Notch pathway in podocytes plays a role in the development of glomerular disease. Nat. Med..

[82] Chen A., Feng Y., Lai H., Ju W., Li Z., Li Y., Wang A., Hong Q., Zhong F., Wei C. (2020). Soluble RARRES1 induces podocyte apoptosis to promote glomerular disease progression. J. Clin. Investig..

[83] Erekat N.S. (2022). Programmed Cell Death in Diabetic Nephropathy: A Review of Apoptosis, Autophagy, and Necroptosis. Med. Sci. Monit..

[84] Gil C.L., Hooker E., Larrivee B. (2021). Diabetic Kidney Disease, Endothelial Damage, and Podocyte-Endothelial Crosstalk. Kidney Med..

[85] Eremina V., Sood M., Haigh J., Nagy A., Lajoie G., Ferrara N., Gerber H.P., Kikkawa Y., Miner J.H., Quaggin S.E. (2003). Glomerular-specific alterations of VEGF-A expression lead to distinct congenital and acquired renal diseases. J. Clin. Investig..

[86] Veron D., Villegas G., Aggarwal P.K., Bertuccio C., Jimenez J., Velazquez H., Reidy K., Abrahamson D.R., Moeckel G., Kashgarian M. (2012). Acute podocyte vascular endothelial growth factor (VEGF-A) knockdown disrupts alphaVbeta3 integrin signaling in the glomerulus. PLoS ONE.

[87] Sharma B., Singh N. (2010). Pitavastatin and 4′-hydroxy-3′-methoxyacetophenone (HMAP) reduce cognitive dysfunction in vascular dementia during experimental diabetes. Curr. Neurovascular Res..

[88] Janelidze S., Hertze J., Nagga K., Nilsson K., Nilsson C., Swedish Bio F.S.G., Wennstrom M., van Westen D., Blennow K., Zetterberg H. (2017). Increased blood-brain barrier permeability is associated with dementia and diabetes but not amyloid pathology or APOE genotype. Neurobiol. Aging.

[89] Stranahan A.M., Hao S., Dey A., Yu X., Baban B. (2016). Blood-brain barrier breakdown promotes macrophage infiltration and cognitive impairment in leptin receptor-deficient mice. J. Cereb. Blood Flow. Metab..

[90] Lacy M.E., Gilsanz P., Eng C., Beeri M.S., Karter A.J., Whitmer R.A. (2020). Severe Hypoglycemia and Cognitive Function in Older Adults with Type 1 Diabetes: The Study of Longevity in Diabetes (SOLID). Diabetes Care.

[91] Kodl C.T., Seaquist E.R. (2008). Cognitive dysfunction and diabetes mellitus. Endocr. Rev..

[92] Luchsinger J.A., Tang M.X., Stern Y., Shea S., Mayeux R. (2001). Diabetes mellitus and risk of Alzheimer’s disease and dementia with stroke in a multiethnic cohort. Am. J. Epidemiol..

[93] Rom S., Zuluaga-Ramirez V., Gajghate S., Seliga A., Winfield M., Heldt N.A., Kolpakov M.A., Bashkirova Y.V., Sabri A.K., Persidsky Y. (2019). Hyperglycemia-Driven Neuroinflammation Compromises BBB Leading to Memory Loss in Both Diabetes Mellitus (DM) Type 1 and Type 2 Mouse Models. Mol. Neurobiol..

[94] Giannini C., Dyck P.J. (1995). Basement membrane reduplication and pericyte degeneration precede development of diabetic polyneuropathy and are associated with its severity. Ann. Neurol..

[95] Lin L., Wu Y., Chen Z., Huang L., Wang L., Liu L. (2021). Severe Hypoglycemia Contributing to Cognitive Dysfunction in Diabetic Mice Is Associated with Pericyte and Blood-Brain Barrier Dysfunction. Front. Aging Neurosci..

[96] Price T.O., Eranki V., Banks W.A., Ercal N., Shah G.N. (2012). Topiramate treatment protects blood-brain barrier pericytes from hyperglycemia-induced oxidative damage in diabetic mice. Endocrinology.

[97] Liu Y., Zhang H., Wang S., Guo Y., Fang X., Zheng B., Gao W., Yu H., Chen Z., Roman R.J. (2021). Reduced pericyte and tight junction coverage in old diabetic rats are associated with hyperglycemia-induced cerebrovascular pericyte dysfunction. Am. J. Physiol. Heart Circ. Physiol..

[98] Shah G.N., Price T.O., Banks W.A., Morofuji Y., Kovac A., Ercal N., Sorenson C.M., Shin E.S., Sheibani N. (2013). Pharmacological inhibition of mitochondrial carbonic anhydrases protects mouse cerebral pericytes from high glucose-induced oxidative stress and apoptosis. J. Pharmacol. Exp. Ther..

[99] Price T.O., Sheibani N., Shah G.N. (2017). Regulation of high glucose-induced apoptosis of brain pericytes by mitochondrial CA VA: A specific target for prevention of diabetic cerebrovascular pathology. Biochim. Biophys. Acta Mol. Basis Dis..

[100] Patrick P., Price T.O., Diogo A.L., Sheibani N., Banks W.A., Shah G.N. (2015). Topiramate Protects Pericytes from Glucotoxicity: Role for Mitochondrial CA VA in Cerebromicrovascular Disease in Diabetes. J. Endocrinol. Diabetes.

[101] May J.M., Jayagopal A., Qu Z.C., Parker W.H. (2014). Ascorbic acid prevents high glucose-induced apoptosis in human brain pericytes. Biochem. Biophys. Res. Commun..

[102] Kannel W.B., Hjortland M., Castelli W.P. (1974). Role of diabetes in congestive heart failure: The Framingham study. Am. J. Cardiol..

[103] Stamler J., Vaccaro O., Neaton J.D., Wentworth D. (1993). Diabetes, other risk factors, and 12-yr cardiovascular mortality for men screened in the Multiple Risk Factor Intervention Trial. Diabetes Care.

[104] Yoon Y.S., Uchida S., Masuo O., Cejna M., Park J.S., Gwon H.C., Kirchmair R., Bahlman F., Walter D., Curry C. (2005). Progressive attenuation of myocardial vascular endothelial growth factor expression is a seminal event in diabetic cardiomyopathy: Restoration of microvascular homeostasis and recovery of cardiac function in diabetic cardiomyopathy after replenishment of local vascular endothelial growth factor. Circulation.

[105] Wakisaka M., Kamouchi M., Kitazono T. (2019). Lessons from the Trials for the Desirable Effects of Sodium Glucose Co-Transporter 2 Inhibitors on Diabetic Cardiovascular Events and Renal Dysfunction. Int. J. Mol. Sci..

[106] Regan T.J., Lyons M.M., Ahmed S.S., Levinson G.E., Oldewurtel H.A., Ahmad M.R., Haider B. (1977). Evidence for cardiomyopathy in familial diabetes mellitus. J. Clin. Investig..

[107] Kawaguchi M., Techigawara M., Ishihata T., Asakura T., Saito F., Maehara K., Maruyama Y. (1997). A comparison of ultrastructural changes on endomyocardial biopsy specimens obtained from patients with diabetes mellitus with and without hypertension. Heart Vessel..

[108] Hinkel R., Howe A., Renner S., Ng J., Lee S., Klett K., Kaczmarek V., Moretti A., Laugwitz K.L., Skroblin P. (2017). Diabetes Mellitus-Induced Microvascular Destabilization in the Myocardium. J. Am. Coll. Cardiol..

[109] Jack C.R., Bennett D.A., Blennow K., Carrillo M.C., Dunn B., Haeberlein S.B., Holtzman D.M., Jagust W., Jessen F., Karlawish J. (2018). NIA-AA Research Framework: Toward a biological definition of Alzheimer’s disease. Alzheimers Dement..

[110] Sweeney M.D., Sagare A.P., Zlokovic B.V. (2018). Blood-brain barrier breakdown in Alzheimer disease and other neurodegenerative disorders. Nat. Rev. Neurol..

[111] Iadecola C., Duering M., Hachinski V., Joutel A., Pendlebury S.T., Schneider J.A., Dichgans M. (2019). Vascular Cognitive Impairment and Dementia: JACC Scientific Expert Panel. J. Am. Coll. Cardiol..

[112] Sagare A.P., Bell R.D., Zhao Z., Ma Q., Winkler E.A., Ramanathan A., Zlokovic B.V. (2013). Pericyte loss influences Alzheimer-like neurodegeneration in mice. Nat. Commun..

[113] Ding R., Hase Y., Ameen-Ali K.E., Ndung’u M., Stevenson W., Barsby J., Gourlay R., Akinyemi T., Akinyemi R., Uemura M.T. (2020). Loss of capillary pericytes and the blood-brain barrier in white matter in poststroke and vascular dementias and Alzheimer’s disease. Brain Pathol..

[114] Montagne A., Nikolakopoulou A.M., Zhao Z., Sagare A.P., Si G., Lazic D., Barnes S.R., Daianu M., Ramanathan A., Go A. (2018). Pericyte degeneration causes white matter dysfunction in the mouse central nervous system. Nat. Med..

[115] Miners J.S., Schulz I., Love S. (2018). Differing associations between Abeta accumulation, hypoperfusion, blood-brain barrier dysfunction and loss of PDGFRB pericyte marker in the precuneus and parietal white matter in Alzheimer’s disease. J. Cereb. Blood Flow. Metab..

[116] Sengillo J.D., Winkler E.A., Walker C.T., Sullivan J.S., Johnson M., Zlokovic B.V. (2013). Deficiency in mural vascular cells coincides with blood-brain barrier disruption in Alzheimer’s disease. Brain Pathol..

[117] Shi H., Koronyo Y., Rentsendorj A., Regis G.C., Sheyn J., Fuchs D.T., Kramerov A.A., Ljubimov A.V., Dumitrascu O.M., Rodriguez A.R. (2020). Identification of early pericyte loss and vascular amyloidosis in Alzheimer’s disease retina. Acta Neuropathol..

[118] Janota C.S., Brites D., Lemere C.A., Brito M.A. (2015). Glio-vascular changes during ageing in wild-type and Alzheimer’s disease-like APP/PS1 mice. Brain Res..

[119] Wu Q., Yuan X., Bai J., Han R., Li Z., Zhang H., Xiu R. (2019). MicroRNA-181a protects against pericyte apoptosis via directly targeting FOXO1: Implication for ameliorated cognitive deficits in APP/PS1 mice. Aging.

[120] Shi H., Koronyo Y., Fuchs D.T., Sheyn J., Wawrowsky K., Lahiri S., Black K.L., Koronyo-Hamaoui M. (2020). Retinal capillary degeneration and blood-retinal barrier disruption in murine models of Alzheimer’s disease. Acta Neuropathol. Commun..

[121] Nortley R., Korte N., Izquierdo P., Hirunpattarasilp C., Mishra A., Jaunmuktane Z., Kyrargyri V., Pfeiffer T., Khennouf L., Madry C. (2019). Amyloid beta oligomers constrict human capillaries in Alzheimer’s disease via signaling to pericytes. Science.

[122] Procter T.V., Williams A., Montagne A. (2021). Interplay between Brain Pericytes and Endothelial Cells in Dementia. Am. J. Pathol..

[123] Hellstrom M., Gerhardt H., Kalen M., Li X., Eriksson U., Wolburg H., Betsholtz C. (2001). Lack of pericytes leads to endothelial hyperplasia and abnormal vascular morphogenesis. J. Cell Biol..

[124] Tachibana M., Yamazaki Y., Liu C.C., Bu G., Kanekiyo T. (2018). Pericyte implantation in the brain enhances cerebral blood flow and reduces amyloid-beta pathology in amyloid model mice. Exp. Neurol..

[125] Kiernan M.C., Vucic S., Cheah B.C., Turner M.R., Eisen A., Hardiman O., Burrell J.R., Zoing M.C. (2011). Amyotrophic lateral sclerosis. Lancet.

[126] Al-Chalabi A., Hardiman O. (2013). The epidemiology of ALS: A conspiracy of genes, environment and time. Nat. Rev. Neurol..

[127] Sasaki S. (2015). Alterations of the blood-spinal cord barrier in sporadic amyotrophic lateral sclerosis. Neuropathology.

[128] Cao M.C., Cawston E.E., Chen G., Brooks C., Douwes J., McLean D., Graham E.S., Dragunow M., Scotter E.L. (2022). Serum biomarkers of neuroinflammation and blood-brain barrier leakage in amyotrophic lateral sclerosis. BMC Neurol..

[129] Coatti G.C., Cavacana N., Zatz M. (2019). The Role of Pericytes in Amyotrophic Lateral Sclerosis. Adv. Exp. Med. Biol..

[130] Yamadera M., Fujimura H., Inoue K., Toyooka K., Mori C., Hirano H., Sakoda S. (2015). Microvascular disturbance with decreased pericyte coverage is prominent in the ventral horn of patients with amyotrophic lateral sclerosis. Amyotroph. Lateral Scler. Front. Degener..

[131] Winkler E.A., Sengillo J.D., Sullivan J.S., Henkel J.S., Appel S.H., Zlokovic B.V. (2013). Blood-spinal cord barrier breakdown and pericyte reductions in amyotrophic lateral sclerosis. Acta Neuropathol..

[132] Saul J., Hutchins E., Reiman R., Saul M., Ostrow L.W., Harris B.T., Van Keuren-Jensen K., Bowser R., Bakkar N. (2020). Global alterations to the choroid plexus blood-CSF barrier in amyotrophic lateral sclerosis. Acta Neuropathol. Commun..

[133] Garbuzova-Davis S., Boccio K.J., Llauget A., Shell R., Hailu S., Mustafa H., Ehrhart J., Sanberg P.R., Appel S.H., Borlongan C.V. (2021). Beneficial Effects of Transplanted Human Bone Marrow Endothelial Progenitors on Functional and Cellular Components of Blood-Spinal Cord Barrier in ALS Mice. Eneuro.

[134] Coatti G.C., Frangini M., Valadares M.C., Gomes J.P., Lima N.O., Cavacana N., Assoni A.F., Pelatti M.V., Birbrair A., de Lima A.C.P. (2017). Pericytes Extend Survival of ALS SOD1 Mice and Induce the Expression of Antioxidant Enzymes in the Murine Model and in IPSCs Derived Neuronal Cells from an ALS Patient. Stem Cell Rev. Rep..

[135] Dolin H.H., Papadimos T.J., Stepkowski S., Chen X., Pan Z.K. (2018). A Novel Combination of Biomarkers to Herald the Onset of Sepsis Prior to the Manifestation of Symptoms. Shock.

[136] Hotchkiss R.S., Moldawer L.L., Opal S.M., Reinhart K., Turnbull I.R., Vincent J.L. (2016). Sepsis and septic shock. Nat. Rev. Dis. Primers.

[137] Stasi A., Franzin R., Divella C., Gesualdo L., Stallone G., Castellano G. (2020). Double Labeling of PDGFR-beta and alpha-SMA in Swine Models of Acute Kidney Injury to Detect Pericyte-to-Myofibroblast Transdifferentation as Early Marker of Fibrosis. Bio Protoc..

[138] Nishioku T., Dohgu S., Takata F., Eto T., Ishikawa N., Kodama K.B., Nakagawa S., Yamauchi A., Kataoka Y. (2009). Detachment of brain pericytes from the basal lamina is involved in disruption of the blood-brain barrier caused by lipopolysaccharide-induced sepsis in mice. Cell Mol. Neurobiol..

[139] Barichello T., Generoso J.S., Collodel A., Petronilho F., Dal-Pizzol F. (2021). The blood-brain barrier dysfunction in sepsis. Tissue Barriers.

[140] Armulik A., Genove G., Mae M., Nisancioglu M.H., Wallgard E., Niaudet C., He L., Norlin J., Lindblom P., Strittmatter K. (2010). Pericytes regulate the blood-brain barrier. Nature.

[141] Zeng H., He X., Tuo Q.H., Liao D.F., Zhang G.Q., Chen J.X. (2016). LPS causes pericyte loss and microvascular dysfunction via disruption of Sirt3/angiopoietins/Tie-2 and HIF-2alpha/Notch3 pathways. Sci. Rep..

[142] Zhang Z.S., Liu Y.Y., He S.S., Bao D.Q., Wang H.C., Zhang J., Peng X.Y., Zang J.T., Zhu Y., Wu Y. (2023). Pericytes protect rats and mice from sepsis-induced injuries by maintaining vascular reactivity and barrier function: Implication of miRNAs and microvesicles. Mil. Med. Res..

[143] Parikh S.M., Mammoto T., Schultz A., Yuan H.T., Christiani D., Karumanchi S.A., Sukhatme V.P. (2006). Excess circulating angiopoietin-2 may contribute to pulmonary vascular leak in sepsis in humans. PLoS Med..

[144] Ziegler T., Horstkotte J., Schwab C., Pfetsch V., Weinmann K., Dietzel S., Rohwedder I., Hinkel R., Gross L., Lee S. (2013). Angiopoietin 2 mediates microvascular and hemodynamic alterations in sepsis. J. Clin. Investig..

[145] Zhou H., Zheng D., Wang H., Wu Y., Peng X., Li Q., Li T., Liu L. (2021). The protective effects of pericyte-derived microvesicles on vascular endothelial functions via CTGF delivery in sepsis. Cell Commun. Signal..

[146] Lindl K.A., Marks D.R., Kolson D.L., Jordan-Sciutto K.L. (2010). HIV-associated neurocognitive disorder: Pathogenesis and therapeutic opportunities. J. Neuroimmune Pharmacol..

[147] Castro V., Bertrand L., Luethen M., Dabrowski S., Lombardi J., Morgan L., Sharova N., Stevenson M., Blasig I.E., Toborek M. (2016). Occludin controls HIV transcription in brain pericytes via regulation of SIRT-1 activation. FASEB J..

[148] Nakagawa S., Castro V., Toborek M. (2012). Infection of human pericytes by HIV-1 disrupts the integrity of the blood-brain barrier. J. Cell Mol. Med..

[149] Torices S., Roberts S.A., Park M., Malhotra A., Toborek M. (2020). Occludin, caveolin-1, and Alix form a multi-protein complex and regulate HIV-1 infection of brain pericytes. FASEB J..

[150] Persidsky Y., Hill J., Zhang M., Dykstra H., Winfield M., Reichenbach N.L., Potula R., Mukherjee A., Ramirez S.H., Rom S. (2016). Dysfunction of brain pericytes in chronic neuroinflammation. J. Cereb. Blood Flow. Metab..

[151] Cho H.J., Kuo A.M., Bertrand L., Toborek M. (2017). HIV Alters Gap Junction-Mediated Intercellular Communication in Human Brain Pericytes. Front. Mol. Neurosci..

[152] Naranjo O., Torices S., Clifford P.R., Daftari M.T., Osborne O.M., Fattakhov N., Toborek M. (2022). Pericyte infection by HIV-1: A fatal attraction. Retrovirology.

[153] Niu F., Yao H., Zhang W., Sutliff R.L., Buch S. (2014). Tat 101-mediated enhancement of brain pericyte migration involves platelet-derived growth factor subunit B homodimer: Implications for human immunodeficiency virus-associated neurocognitive disorders. J. Neurosci..

[154] Bohannon D.G., Ko A., Filipowicz A.R., Kuroda M.J., Kim W.K. (2019). Dysregulation of sonic hedgehog pathway and pericytes in the brain after lentiviral infection. J. Neuroinflamm..

[155] Niu F., Yao H., Liao K., Buch S. (2015). HIV Tat 101-mediated loss of pericytes at the blood-brain barrier involves PDGF-BB. Ther. Targets Neurol. Dis..

[156] Burkert F.R., Lanser L., Bellmann-Weiler R., Weiss G. (2021). Coronavirus Disease 2019: Clinics, Treatment, and Prevention. Front. Microbiol..

[157] Avolio E., Carrabba M., Milligan R., Kavanagh Williamson M., Beltrami A.P., Gupta K., Elvers K.T., Gamez M., Foster R.R., Gillespie K. (2021). The SARS-CoV-2 Spike protein disrupts human cardiac pericytes function through CD147 receptor-mediated signalling: A potential non-infective mechanism of COVID-19 microvascular disease. Clin. Sci..

[158] Bocci M., Oudenaarden C., Saenz-Sarda X., Simren J., Eden A., Sjolund J., Moller C., Gisslen M., Zetterberg H., Englund E. (2021). Infection of Brain Pericytes Underlying Neuropathology of COVID-19 Patients. Int. J. Mol. Sci..

[159] Khan A.O., Reyat J.S., Hill H., Bourne J.H., Colicchia M., Newby M.L., Allen J.D., Crispin M., Youd E., Murray P.G. (2022). Preferential uptake of SARS-CoV-2 by pericytes potentiates vascular damage and permeability in an organoid model of the microvasculature. Cardiovasc. Res..

[160] Das M., Chung M.K. (2023). Cardiac Pericytes: Underappreciated Targets of SARS-CoV-2. JACC Basic Transl. Sci..

[161] Nicin L., Abplanalp W.T., Mellentin H., Kattih B., Tombor L., John D., Schmitto J.D., Heineke J., Emrich F., Arsalan M. (2020). Cell type-specific expression of the putative SARS-CoV-2 receptor ACE2 in human hearts. Eur. Heart J..

[162] Brumback B.D., Dmytrenko O., Robinson A.N., Bailey A.L., Ma P., Liu J., Hicks S.C., Ng S., Li G., Zhang D.M. (2023). Human Cardiac Pericytes Are Susceptible to SARS-CoV-2 Infection. JACC Basic Transl. Sci..

[163] Robinson F.A., Mihealsick R.P., Wagener B.M., Hanna P., Poston M.D., Efimov I.R., Shivkumar K., Hoover D.B. (2020). Role of angiotensin-converting enzyme 2 and pericytes in cardiac complications of COVID-19 infection. Am. J. Physiol. Heart Circ. Physiol..

[164] Daems M., Liesenborghs L., Boudewijns R., Simmonds S.J., Kraisin S., Van Wauwe J., Cuijpers I., Raman J., Geuens N., Buyten T.V. (2022). SARS-CoV-2 infection causes prolonged cardiomyocyte swelling and inhibition of HIF1alpha translocation in an animal model COVID-19. Front. Cardiovasc. Med..

[165] Muhl L., He L., Sun Y., Andaloussi Mae M., Pietila R., Liu J., Genove G., Zhang L., Xie Y., Leptidis S. (2022). The SARS-CoV-2 receptor ACE2 is expressed in mouse pericytes but not endothelial cells: Implications for COVID-19 vascular research. Stem Cell Rep..

[166] Miners J.S., Fisher R.A., Love S. (2023). SARS-CoV-2 targets pericytes to restrict blood flow within the brain. Brain.

[167] Khaddaj-Mallat R., Aldib N., Bernard M., Paquette A.S., Ferreira A., Lecordier S., Saghatelyan A., Flamand L., ElAli A. (2021). SARS-CoV-2 deregulates the vascular and immune functions of brain pericytes via Spike protein. Neurobiol. Dis..

[168] Ju J., Su Y., Zhou Y., Wei H., Xu Q. (2022). The SARS-CoV-2 envelope protein disrupts barrier function in an in vitro human blood-brain barrier model. Front. Cell Neurosci..

[169] Cardot-Leccia N., Hubiche T., Dellamonica J., Burel-Vandenbos F., Passeron T. (2020). Pericyte alteration sheds light on micro-vasculopathy in COVID-19 infection. Intensive Care Med..

[170] Jones O.Y., Yeralan S. (2022). Is Long COVID a State of Systemic Pericyte Disarray?. J. Clin. Med..

[171] Uemura M.T., Maki T., Ihara M., Lee V.M.Y., Trojanowski J.Q. (2020). Brain Microvascular Pericytes in Vascular Cognitive Impairment and Dementia. Front. Aging Neurosci..

[172] Cao L., Zhou Y., Chen M., Li L., Zhang W. (2021). Pericytes for Therapeutic Approaches to Ischemic Stroke. Front. Neurosci..

[173] Fernandez-Klett F., Potas J.R., Hilpert D., Blazej K., Radke J., Huck J., Engel O., Stenzel W., Genove G., Priller J. (2013). Early loss of pericytes and perivascular stromal cell-induced scar formation after stroke. J. Cereb. Blood Flow. Metab..

[174] Zhang Y., Zhang X., Wei Q., Leng S., Li C., Han B., Bai Y., Zhang H., Yao H. (2020). Activation of Sigma-1 Receptor Enhanced Pericyte Survival via the Interplay between Apoptosis and Autophagy: Implications for Blood-Brain Barrier Integrity in Stroke. Transl. Stroke Res..

[175] Tsao C.C., Baumann J., Huang S.F., Kindler D., Schroeter A., Kachappilly N., Gassmann M., Rudin M., Ogunshola O.O. (2021). Pericyte hypoxia-inducible factor-1 (HIF-1) drives blood-brain barrier disruption and impacts acute ischemic stroke outcome. Angiogenesis.

[176] Zhou Y.F., Li P.C., Wu J.H., Haslam J.A., Mao L., Xia Y.P., He Q.W., Wang X.X., Lei H., Lan X.L. (2018). Sema3E/PlexinD1 inhibition is a therapeutic strategy for improving cerebral perfusion and restoring functional loss after stroke in aged rats. Neurobiol. Aging.

[177] Ozen I., Roth M., Barbariga M., Gaceb A., Deierborg T., Genove G., Paul G. (2018). Loss of Regulator of G-Protein Signaling 5 Leads to Neurovascular Protection in Stroke. Stroke.

[178] Cai W., Liu H., Zhao J., Chen L.Y., Chen J., Lu Z., Hu X. (2017). Pericytes in Brain Injury and Repair After Ischemic Stroke. Transl. Stroke Res..

[179] Gundersen G.A., Vindedal G.F., Skare O., Nagelhus E.A. (2014). Evidence that pericytes regulate aquaporin-4 polarization in mouse cortical astrocytes. Brain Struct. Funct..

[180] Hori S., Ohtsuki S., Hosoya K., Nakashima E., Terasaki T. (2004). A pericyte-derived angiopoietin-1 multimeric complex induces occludin gene expression in brain capillary endothelial cells through Tie-2 activation in vitro. J. Neurochem..

[181] Sundberg C., Kowanetz M., Brown L.F., Detmar M., Dvorak H.F. (2002). Stable expression of angiopoietin-1 and other markers by cultured pericytes: Phenotypic similarities to a subpopulation of cells in maturing vessels during later stages of angiogenesis in vivo. Lab. Investig..

[182] van Dijk C.G., Nieuweboer F.E., Pei J.Y., Xu Y.J., Burgisser P., van Mulligen E., el Azzouzi H., Duncker D.J., Verhaar M.C., Cheng C. (2015). The complex mural cell: Pericyte function in health and disease. Int. J. Cardiol..

[183] Menon D.K., Schwab K., Wright D.W., Maas A.I. (2010). Demographics and Clinical Assessment Working Group of the International and Interagency Initiative toward Common Data Elements for Research on Traumatic Brain Injury and Psychological Health. Position statement: Definition of traumatic brain injury. Arch. Phys. Med. Rehabil..

[184] Ladak A.A., Enam S.A., Ibrahim M.T. (2019). A Review of the Molecular Mechanisms of Traumatic Brain Injury. World Neurosurg..

[185] Abdul-Muneer P.M., Long M., Conte A.A., Santhakumar V., Pfister B.J. (2017). High Ca(2+) Influx During Traumatic Brain Injury Leads to Caspase-1-Dependent Neuroinflammation and Cell Death. Mol. Neurobiol..

[186] Patel R.K., Prasad N., Kuwar R., Haldar D., Abdul-Muneer P.M. (2017). Transforming growth factor-beta 1 signaling regulates neuroinflammation and apoptosis in mild traumatic brain injury. Brain Behav. Immun..

[187] Dore-Duffy P., Wang S., Mehedi A., Katyshev V., Cleary K., Tapper A., Reynolds C., Ding Y., Zhan P., Rafols J. (2011). Pericyte-mediated vasoconstriction underlies TBI-induced hypoperfusion. Neurol. Res..

[188] Zheng S., Wang C., Lin L., Mu S., Liu H., Hu X., Chen X., Wang S. (2023). TNF-alpha Impairs Pericyte-Mediated Cerebral Microcirculation via the NF-kappaB/iNOS Axis after Experimental Traumatic Brain Injury. J. Neurotrauma.

[189] Ojo J., Eisenbaum M., Shackleton B., Lynch C., Joshi U., Saltiel N., Pearson A., Ringland C., Paris D., Mouzon B. (2021). Mural cell dysfunction leads to altered cerebrovascular tau uptake following repetitive head trauma. Neurobiol. Dis..

[190] Zehendner C.M., Sebastiani A., Hugonnet A., Bischoff F., Luhmann H.J., Thal S.C. (2015). Traumatic brain injury results in rapid pericyte loss followed by reactive pericytosis in the cerebral cortex. Sci. Rep..

[191] Bhowmick S., D’Mello V., Caruso D., Wallerstein A., Abdul-Muneer P.M. (2019). Impairment of pericyte-endothelium crosstalk leads to blood-brain barrier dysfunction following traumatic brain injury. Exp. Neurol..

[192] Wu Y., Wu H., Zeng J., Pluimer B., Dong S., Xie X., Guo X., Ge T., Liang X., Feng S. (2021). Mild traumatic brain injury induces microvascular injury and accelerates Alzheimer-like pathogenesis in mice. Acta Neuropathol. Commun..

[193] Choi Y.K., Maki T., Mandeville E.T., Koh S.H., Hayakawa K., Arai K., Kim Y.M., Whalen M.J., Xing C., Wang X. (2016). Dual effects of carbon monoxide on pericytes and neurogenesis in traumatic brain injury. Nat. Med..

[194] Uzunalli G., Dieterly A.M., Kemet C.M., Weng H.Y., Soepriatna A.H., Goergen C.J., Shinde A.B., Wendt M.K., Lyle L.T. (2019). Dynamic transition of the blood-brain barrier in the development of non-small cell lung cancer brain metastases. Oncotarget.

[195] Kaushik D.K., Bhattacharya A., Lozinski B.M., Wee Yong V. (2021). Pericytes as mediators of infiltration of macrophages in multiple sclerosis. J. Neuroinflamm..

[196] Halliday M.R., Rege S.V., Ma Q., Zhao Z., Miller C.A., Winkler E.A., Zlokovic B.V. (2016). Accelerated pericyte degeneration and blood-brain barrier breakdown in apolipoprotein E4 carriers with Alzheimer’s disease. J. Cereb. Blood Flow. Metab..

[197] Eilam R., Segal M., Malach R., Sela M., Arnon R., Aharoni R. (2018). Astrocyte disruption of neurovascular communication is linked to cortical damage in an animal model of multiple sclerosis. Glia.

[198] Medina-Flores F., Hurtado-Alvarado G., Contis-Montes de Oca A., Lopez-Cervantes S.P., Konigsberg M., Deli M.A., Gomez-Gonzalez B. (2020). Sleep loss disrupts pericyte-brain endothelial cell interactions impairing blood-brain barrier function. Brain Behav. Immun..

[199] Kofler N.M., Cuervo H., Uh M.K., Murtomaki A., Kitajewski J. (2015). Combined deficiency of Notch1 and Notch3 causes pericyte dysfunction, models CADASIL, and results in arteriovenous malformations. Sci. Rep..

[200] Wilkinson-Berka J.L., Babic S., De Gooyer T., Stitt A.W., Jaworski K., Ong L.G., Kelly D.J., Gilbert R.E. (2004). Inhibition of platelet-derived growth factor promotes pericyte loss and angiogenesis in ischemic retinopathy. Am. J. Pathol..

[201] Lin L., Chen Z., Huang C., Wu Y., Huang L., Wang L., Ke S., Liu L. (2023). Mito-TEMPO, a Mitochondria-Targeted Antioxidant, Improves Cognitive Dysfunction due to Hypoglycemia: An Association with Reduced Pericyte Loss and Blood-Brain Barrier Leakage. Mol. Neurobiol..

[202] Blervaque L., Passerieux E., Pomies P., Catteau M., Heraud N., Blaquiere M., Bughin F., Ayoub B., Molinari N., Cristol J.P. (2019). Impaired training-induced angiogenesis process with loss of pericyte-endothelium interactions is associated with an abnormal capillary remodelling in the skeletal muscle of COPD patients. Respir. Res..

[203] Proebstl D., Voisin M.B., Woodfin A., Whiteford J., D’Acquisto F., Jones G.E., Rowe D., Nourshargh S. (2012). Pericytes support neutrophil subendothelial cell crawling and breaching of venular walls in vivo. J. Exp. Med..

[204] Seynhaeve A.L.B., Oostinga D., van Haperen R., Eilken H.M., Adams S., Adams R.H., Ten Hagen T.L.M. (2018). Spatiotemporal endothelial cell—Pericyte association in tumors as shown by high resolution 4D intravital imaging. Sci. Rep..

[205] Seynhaeve A.L.B., Ten Hagen T.L.M. (2021). An adapted dorsal skinfold model used for 4D intravital followed by whole-mount imaging to reveal endothelial cell-pericyte association. Sci. Rep..

[206] Underly R.G., Levy M., Hartmann D.A., Grant R.I., Watson A.N., Shih A.Y. (2017). Pericytes as Inducers of Rapid, Matrix Metalloproteinase-9-Dependent Capillary Damage during Ischemia. J. Neurosci..

[207] Fernandez-Klett F., Brandt L., Fernandez-Zapata C., Abuelnor B., Middeldorp J., Sluijs J.A., Curtis M., Faull R., Harris L.W., Bahn S. (2020). Denser brain capillary network with preserved pericytes in Alzheimer’s disease. Brain Pathol..

[208] Jeffrey D.A., Fontaine J.T., Dabertrand F. (2022). Ex vivo capillary-parenchymal arteriole approach to study brain pericyte physiology. Neurophotonics.

[209] Zhou H.J., Qin L., Jiang Q., Murray K.N., Zhang H., Li B., Lin Q., Graham M., Liu X., Grutzendler J. (2021). Caveolae-mediated Tie2 signaling contributes to CCM pathogenesis in a brain endothelial cell-specific Pdcd10-deficient mouse model. Nat. Commun..

[210] Grant R.I., Hartmann D.A., Underly R.G., Berthiaume A.A., Bhat N.R., Shih A.Y. (2019). Organizational hierarchy and structural diversity of microvascular pericytes in adult mouse cortex. J. Cereb. Blood Flow. Metab..

[211] Miners J.S., Kehoe P.G., Love S., Zetterberg H., Blennow K. (2019). CSF evidence of pericyte damage in Alzheimer’s disease is associated with markers of blood-brain barrier dysfunction and disease pathology. Alzheimers Res. Ther..

[212] Sweeney M.D., Sagare A.P., Pachicano M., Harrington M.G., Joe E., Chui H.C., Schneider L.S., Montagne A., Ringman J.M., Fagan A.M. (2020). A novel sensitive assay for detection of a biomarker of pericyte injury in cerebrospinal fluid. Alzheimers Dement..

[213] Nation D.A., Sweeney M.D., Montagne A., Sagare A.P., D’Orazio L.M., Pachicano M., Sepehrband F., Nelson A.R., Buennagel D.P., Harrington M.G. (2019). Blood-brain barrier breakdown is an early biomarker of human cognitive dysfunction. Nat. Med..

[214] Li X., Pan J., Liu T., Yin W., Miao Q., Zhao Z., Gao Y., Zheng W., Li H., Deng R. (2023). Novel TCF21(high) pericyte subpopulation promotes colorectal cancer metastasis by remodelling perivascular matrix. Gut.

[215] Peisker F., Halder M., Nagai J., Ziegler S., Kaesler N., Hoeft K., Li R., Bindels E.M.J., Kuppe C., Moellmann J. (2022). Mapping the cardiac vascular niche in heart failure. Nat. Commun..

[216] Lu T., Zhang J., Lu S., Yang F., Gan L., Wu X., Song H., Liu S., Xu C., Han D. (2023). Endosialin-positive tumor-derived pericytes promote tumor progression through impeding the infiltration of CD8(+) T cells in clear cell renal cell carcinoma. Cancer Immunol. Immunother..

[217] Jin X., Wang Q., Luo F., Pan J., Lu T., Zhao Y., Zhang X., Xiang E., Zhou C., Huang B. (2023). Single-cell transcriptomic analysis of tumor heterogeneity and intercellular networks in human urothelial carcinoma. Chin. Med. J..

[218] Yang A.C., Vest R.T., Kern F., Lee D.P., Agam M., Maat C.A., Losada P.M., Chen M.B., Schaum N., Khoury N. (2022). A human brain vascular atlas reveals diverse mediators of Alzheimer’s risk. Nature.

[219] Cheng J., Korte N., Nortley R., Sethi H., Tang Y., Attwell D. (2018). Targeting pericytes for therapeutic approaches to neurological disorders. Acta Neuropathol..

